# Viral modulation of cellular RNA alternative splicing: A new key player in virus–host interactions?

**DOI:** 10.1002/wrna.1543

**Published:** 2019-04-29

**Authors:** Simon Boudreault, Patricia Roy, Guy Lemay, Martin Bisaillon

**Affiliations:** ^1^ Département de biochimie, Faculté de médecine et des sciences de la santé Université de Sherbrooke Sherbrooke Québec Canada; ^2^ Département de microbiologie, infectiologie et immunologie, Faculté de médecine Université de Montréal Montréal Québec Canada

**Keywords:** RNA alternative splicing, virus, virus–host interactions

## Abstract

Upon viral infection, a tug of war is triggered between host cells and viruses to maintain/gain control of vital cellular functions, the result of which will ultimately dictate the fate of the host cell. Among these essential cellular functions, alternative splicing (AS) is an important RNA maturation step that allows exons, or parts of exons, and introns to be retained in mature transcripts, thereby expanding proteome diversity and function. AS is widespread in higher eukaryotes, as it is estimated that nearly all genes in humans are alternatively spliced. Recent evidence has shown that upon infection by numerous viruses, the AS landscape of host‐cells is affected. In this review, we summarize recent advances in our understanding of how virus infection impacts the AS of cellular transcripts. We also present various molecular mechanisms allowing viruses to modulate cellular AS. Finally, the functional consequences of these changes in the RNA splicing signatures during virus–host interactions are discussed.

This article is categorized under:

RNA in Disease and Development > RNA in Disease
RNA Processing > Splicing Regulation/Alternative Splicing

## 1 INTRODUCTION

The study of virus–host interaction has been a long and meticulous work to decipher both the impact of viral infection on the host cell, the response of the host cell to viral proteins and RNA, and the complex interplay and cross‐talk between both. Upon infection, a race is established between the virus and its host cell to either gain control of cellular functions, vital for viral replication, or mount an efficient antiviral response to prevent viral spread. The result of this race is either replication for the virus or control of the infection and clearance for the host cell. Upon infection, viral determinants such as double‐stranded RNA (dsRNA) are recognized by PRRs (pattern‐recognition receptors) that trigger a signaling cascade that leads to the production of interferon (IFN). Upon secretion, IFN can act both in a paracrine fashion on uninfected cells to prepare them for infection, or in an autocrine manner to stimulate the infected cell. Upon binding and signaling, IFN leads to the expression of a plethora of interferon‐stimulated genes (ISG) that are effectors of the cellular antiviral response against infection (Fensterl, Chattopadhyay, & Sen, 2015; Sen & Sarkar, 2007). Hence, the transcriptome of the host cell is profoundly impacted by viral infection, with hundreds of genes being rapidly overexpressed in order of magnitude that can climb up to 10,000‐fold.

**Figure 1:**
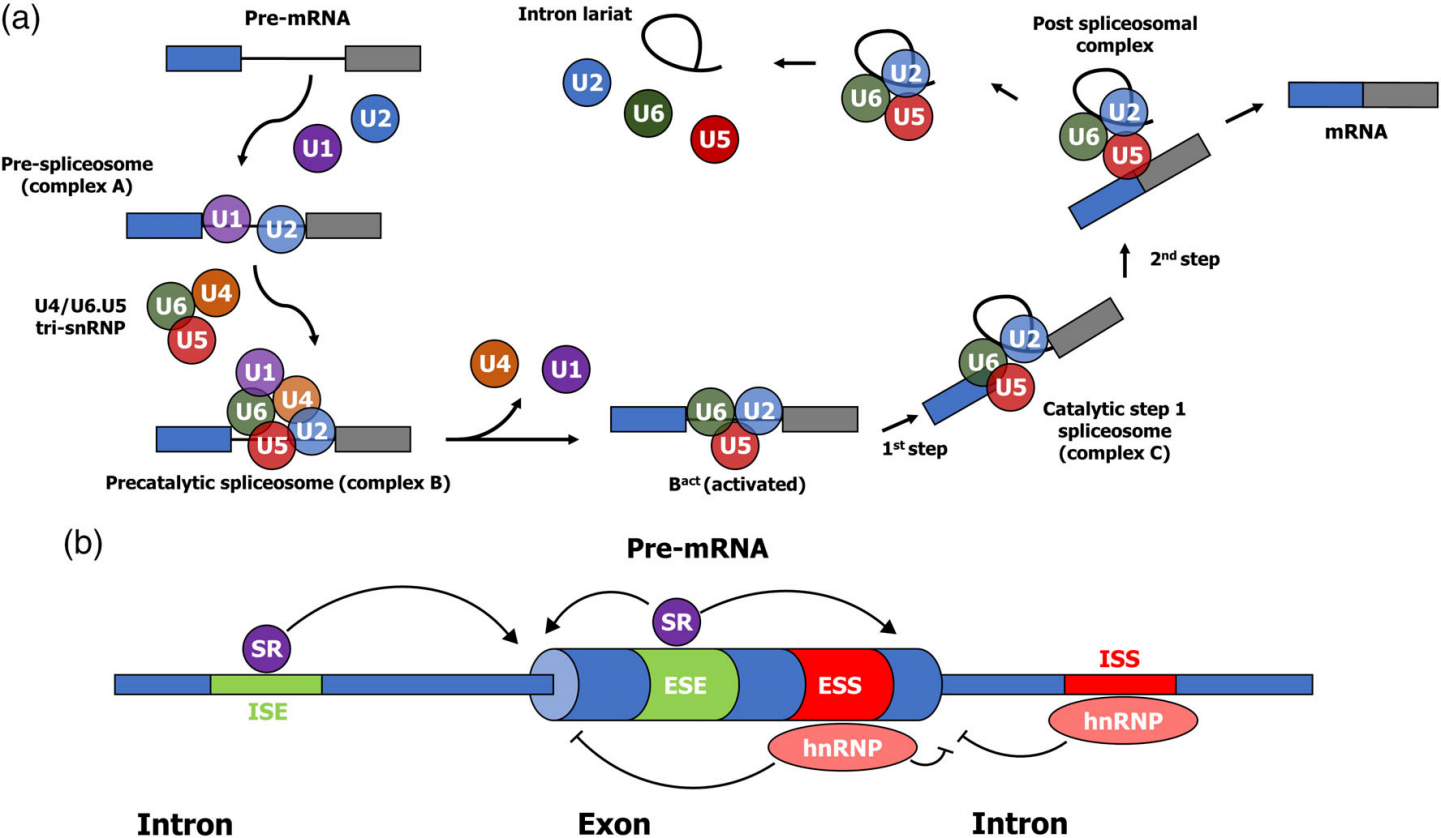
Summary of the splicing reaction and regulatory signals/proteins involved. (a) The cycle of assembly and disassembly of the spliceosome throughout the splicing reaction. The stepwise interaction of the spliceosomal small ribonucleoprotein (snRNP) particles (U1, U2, U4, U4, U5, and U6; colored circles) in the excision of an intron from a pre‐mature RNA (pre‐mRNA) containing two exons (blue and gray) is depicted. The name of the spliceosomal complexes and the two catalytic steps of the reaction are indicated. (b) Positive and negative signals are stabilizing or destabilizing the assembly of the spliceosome on the pre‐mRNA by *cis*‐acting elements. The diagram represents a typical segment of eukaryotic precursor messenger RNA with one exon and the two surrounding introns. Intronic and exonic splicing enhancers (ISE and ESE; in green) are typically bound by factors promoting the splicing reaction from nearby splice sites, such as serine‐arginine repeats (SR) proteins. Intronic and exonic splicing silencers (ISS and ESS; in red) are typically bound by factors inhibiting splicing from nearby splice sites, such as heterogeneous nuclear ribonucleoprotein particle (hnRNP) proteins

Constitutive splicing is a vital RNA maturation step that allows the removal of introns, which are non‐coding sequences, from the pre‐mature transcript. To do so, the spliceosome, a large ribonucleoprotein made of multiple U spliceosomal RNAs and proteins, recognize the splice junction and catalyze two trans‐esterification reactions to excise the intron (Figure 1a) (reviewed in Kim, Goren, & Ast, 2008; Y. Lee & Rio, 2015; Merkhofer, Hu, & Johnson, 2014). Briefly, the first step is the binding of U1 and U2 snRNP to the 5′ splice site and the branch site, respectively. Then, the U4/U6.U5 tri‐snRNP is recruited. Upon structural reorganization, U1 and U4 snRNP are released, leading to an activated B complex. The branch site becomes close in proximity to the 5′ splice site through the new connection between U6 and U2 snRNP. In the first step, the 2′ hydroxyl of the branch site performs a nucleophilic attack on the 5′ phosphate of the first nucleotide of the intron (5′ splice site), usually a guanosine. The reaction breaks the bond between the exon and the intron, forming a new one with the branch site and the intron. In the second catalytic reaction, the newly free 3’ OH group attacks the last intronic nucleotide, also usually a guanosine. This links the two exons together, thereby releasing the intron lariat.

**Figure 2:**
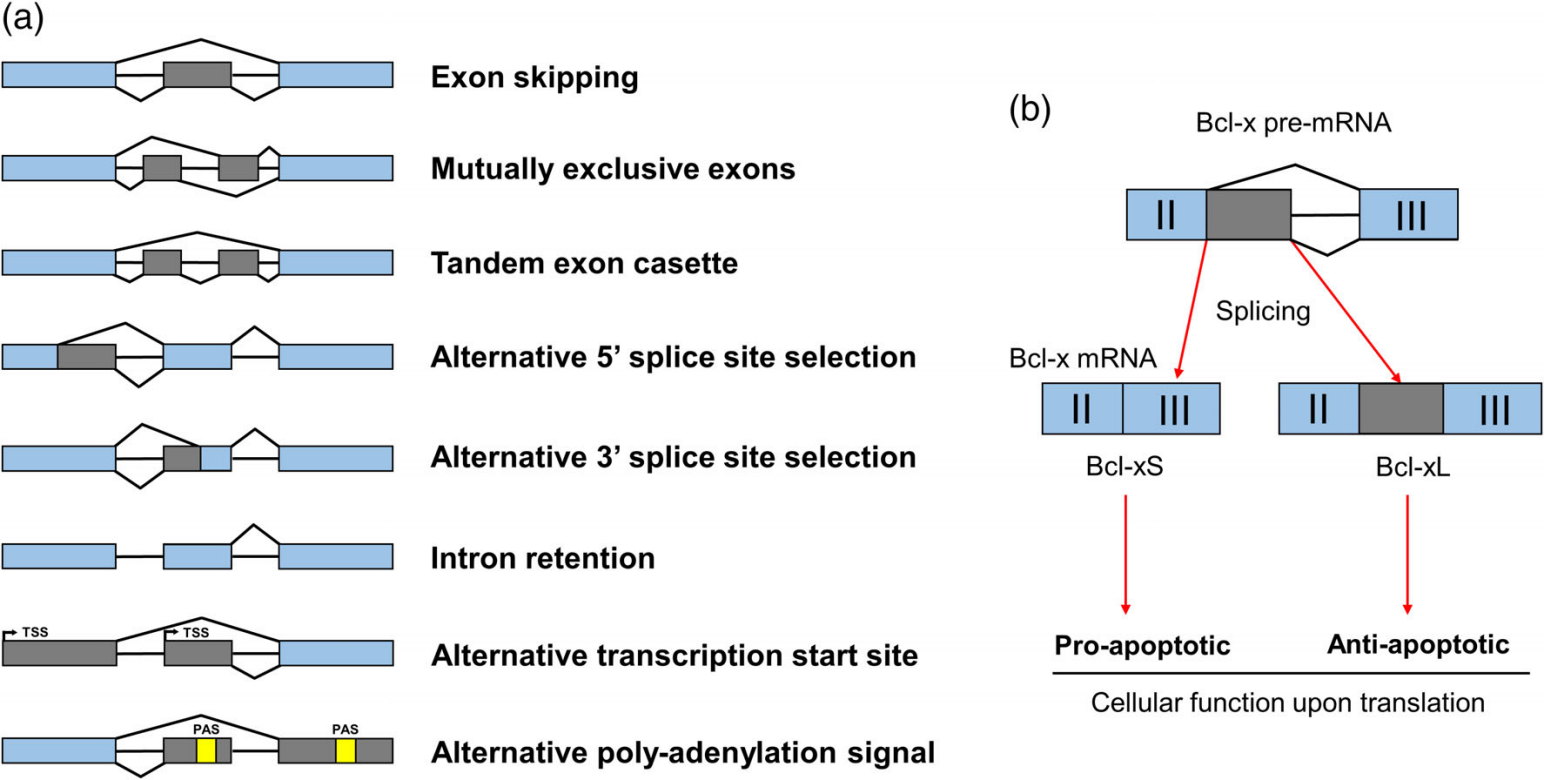
Summary of the different types of AS events and the biological role of AS. (a) Exon skipping, mutually exclusive exon and tandem exon cassette allow selective removal of complete exons from the mature RNA. Alternative 5′ and 3′ splice site selection allows removal of a part of an exon, either in 5′ or 3′ using the intron as the reference. An intron might be kept in the mature RNA, leading to intron retention. Gray boxes represent regions that are alternatively spliced; blue boxes represent regions that are always conserved in the mature mRNA; yellow boxes represent poly‐adenylation signals (PAS). TSS: Transcription start site. (b) The Bcl‐x pre‐mRNA is depicted, with the gray region (alternative 5′ splice site) being spliced in or out to give rise to the short (Bcl‐xS) or long (Bcl‐xL) isoforms. The former produces a pro‐apoptotic protein, and the latter an anti‐apoptotic one, underlining the importance of AS for the regulation of biological activities of proteins

To complement this constitutive removal of introns, some exons, parts of exons, or introns can also be retained in the mature transcript by a process which is called alternative splicing (AS). AS arises from stimulatory and inhibitory signals coming from multiple splicing factors near weak splice sites, either helping the spliceosome to assemble at this location, or destabilizing it and giving rise to a mixed population of mature mRNAs (Figure 1b) (reviewed in Wang et al., 2015; Woodley & Valcárcel, 2002). Enhancing factors, such as SR proteins, bind to either intronic or exonic splicing enhancers (ISE and ESE respectively); inhibitory factors, such as hnRNP proteins, rather bind intronic or exonic splicing silencers (ISS and ESS respectively). AS is widespread in higher eukaryotes, as it is estimated that nearly all genes in humans are alternatively spliced (Wang et al., 2008). Many different types of AS events exist and are summarized in Figure 2a. Mainly, exon skipping, mutually exclusive exon, and tandem exon cassette all lead to different layouts of the same exons in the mature mRNA. Alternative 5′ and 3′ splice site selection can also lead to the removal of specific portions of exons. An intron might also not be properly recognized by the spliceosome and be kept in the mature RNA, leading to intron retention. Furthermore, different transcription start sites (TSS) might be used to initiate transcription, leading to alternative 5′ exons. Finally, poly‐adenylation signals (PAS) might be differentially present in the spliced regions, promoting the use of different polyadenylation sites in isoforms from the same pre‐mRNA. Functionally, AS allows a fine‐tuning of the activity of encoded proteins by removing coding parts corresponding to domains, localization signals, or by introducing frameshifts and premature stop codons. A well‐known example is the Bcl‐x transcript, which upon splicing, can produce either a short pro‐apoptotic protein or a longer anti‐apoptotic protein upon differential usage of a 5′ splice site (Wilhelm, Pellay, Benecke, & Bell, 2008) (Figure 2b). Other examples of alternatively spliced genes that are relevant to the replication of viruses are also well described, such as the Coxsackie virus and adenovirus receptor (CAR) gene. The *CXADR* gene produces a pre‐mRNA that can be matured to generate the full‐length CAR protein, and also a spliced isoform which lacks the transmembrane domain and is thus soluble. This isoform can prevent the binding of the virus to the complete receptor upon secretion (Dörner, Xiong, Couch, Yajima, & Knowlton, 2004). In another example, the murine *IRAK2* gene produces four well‐described isoforms, including two isoforms which can stimulate the NF‐κB pathway, and two other isoforms which act as dominant‐negative forms (Hardy & O'Neill, 2004). Lastly, the *TRAF3* gene, involved in signal transduction of members of the tumor necrosis factor (TNF) family and vital for the activation of NF‐κB and immune responses, possesses multiple alternatively spliced mRNA transcripts (van Eyndhoven et al., 1998). Full‐length TRAF3 protein is unable to activate the NF‐κB pathway but can potentiate the signaling induced by alternatively‐spliced TRAF3 proteins (van Eyndhoven, Gamper, Cho, Mackus, & Lederman, 1999). These few examples only partially reflect the importance of AS in immune response and their potential involvement in virus–host interactions.

More than 40 years ago, the discovery of RNA sequences removed from mature RNA in adenovirus transcripts allowed the identification of splicing (Berget, Moore, & Sharp, 1977; Chow, Gelinas, Broker, & Roberts, 1977). Since then, the importance of AS for some viruses, such as DNA viruses, has become well understood. Human papillomavirus (HPV), human immunodeficiency virus (HIV), and adenoviruses all necessitate the cellular splicing machinery to efficiently splice their mRNAs (for an exhaustive review on how viruses hijack the splicing machinery for their replication, see Meyer, 2016). For these viruses, splicing allows a relatively small genome to encode a broader range of functionally diverse proteins. However, the impact of viral infection on the cellular AS landscape was not studied thoroughly until very recently. The increased affordability and versatility of high‐throughput sequencing technologies, such as RNA‐sequencing, allowed scientists to probe the transcriptome of cells under specific conditions with a depth and width that was not possible using previous techniques. However, technical challenges linked to the study of these virus‐induced changes in cellular AS have been reported (Ashraf, Benoit‐Pilven, Lacroix, Navratil, & Naffakh, 2019). In the present review, we examine the putative impact of these changes in AS on the interplay between viruses and host cells. A complete overview of known examples found in the literature and mechanisms allowing viruses to modulate cellular AS will be initially presented. Then, the impact of this modulation on virus–host interaction, and perspectives regarding the importance of studying this modulation will be discussed.

## 2 CONTRIBUTION OF HIGH‐THROUGHPUT APPROACHES

The advent of high‐throughput sequencing approaches allowed scientists to probe the whole transcriptome of cells under different conditions, such as during viral infection. The initial aim of these studies was to depict the global portrait of the changes in gene expression triggered by infection. However, some of the studies performed also focused on the impact on mRNA maturation processes, such as AS. Although depicting the global portrait of all AS events being modulated during infection, transcriptomic studies alone are frequently only the starting point in assessing changes in AS. Subsequent validation of AS events using PCR‐based technique and more in‐depth mechanistic studies are needed to strengthen the conclusions. In the next sections, examples regarding DNA viruses will first be discussed, then RNA viruses will be addressed, and finally plant RNA viruses will be examined and are summarized in Table 1.

**Table 1:** List of viruses shown to modulate AS in transcriptomic studies

Virus	Family	Genome	Validation of results	Additional details	References
**HCMV**	*Herpesviridae*	dsDNA	++	Validation using HSV‐2	Batra et al. (2016)
**HSV‐1**	*Herpesviridae*	dsDNA	−	Solely transcriptomic	Hu et al. (2016)
**EBV**	*Herpesviridae*	dsDNA	− + +	EBER1 and EBER2 expression EBNA1 expression; high‐throughput RT‐PCR Limited screen using microarray chips	Pimienta et al. (2015); Boudreault, Armero, Scott, Perreault, and Bisaillon (2019); Homa et al. (2013)
**HTLV‐1**	*Herpesviridae*	dsDNA	++	Limited screen using microarray chips	Thénoz et al. (2014)
**HPV**	*Papillomaviridae*	dsDNA	+	Expression of E6	Xu et al. (2016)
**Mammalian reovirus**	*Reoviridae*	dsRNA	++ +	–	Boudreault et al. (2016); Rivera‐Serrano, Fritch, Scholl, and Sherry (2017)
**Avian reovirus**	*Reoviridae*	dsRNA	−	Solely transcriptomic	Niu, Wang, Li, Zhang, and Wu (2017)
**FMDV**	*Picornaviridae*	(+)ssRNA	+	–	Han et al. (2018)
**Zika virus**	*Flaviviridae*	(+)ssRNA	−	Solely transcriptomic	Hu et al. (2017)
**Dengue virus**	*Flaviviridae*	(+)ssRNA	++	–	Sessions et al. (2013)
**Lentivirus**	*Retroviridae*	ssRNA‐RT	++	–	Cesana et al. (2012); Moiani et al. (2012)
**Reticuloendotheliosis virus**	*Retroviridae*	ssRNA‐RT	−	Solely transcriptomic	Gao, Zhai, Dang, and Zheng (2018)
**Influenza A virus**	*Orthomyxoviridae*	(−)ssRNA	+ +	–	Thompson et al. (2018); Fabozzi et al. (2018)
**Panicum mosaic virus**	*Tombusviridae*	(+)ssRNA	+	–	Mandadi and Scholthof (2015)
**Cucumber mosaic virus**	*Bromoviridae*	(+)ssRNA	−	Solely transcriptomic	Zhu, Li, and Zheng (2018)
**Bean common mosaic virus**	*Potyviridae*	(+)ssRNA	−	Solely transcriptomic	Martin, Singh, Hill, Whitham, and Cannon (2016)
**PSTVd**	*Pospiviroidae*	ssRNA	−	Viroid; solely transcriptomic	Zheng, Wang, Ding, and Fei (2017)

### 2.1 DNA viruses

The *Herpesviridae* family, which possesses linear dsDNA genomes, has been the most studied for its ability to modulate AS. For example, the human cytomegalovirus (HCMV) infection triggers the overexpression of the RNA‐binding protein CPEB1, which leads to a change in cellular AS (Batra et al., 2016). Overexpression of CPEB1 in non‐infected cells mimicked the modifications seen in HCMV‐infected cells, hence proving the involvement of CPEB1 in AS. Moreover, this change in AS was also observed following herpes simplex virus 2 (HSV‐2) infection, further solidifying the results and pointing towards a possible conserved modulation of cellular AS for *Herpesviridae* members. A study focusing on transcriptomic changes during herpes simplex virus 1 (HSV‐1) also showed modulation of cellular AS that mainly impacted genes involved in the cell cycle; this study included an RT‐PCR validation of the changes in AS, although for only four genes (Hu et al., 2016). Moreover, the expression in cells of EBER1 and EBER2, two long non‐coding RNAs localized to the nucleus from the Epstein–Barr virus (EBV), another *Herpesviridae*, induced changes in the AS profiles of transcripts linked to EBV oncogenic potential (Pimienta et al., 2015). This is quite interesting, as EBV is both a latent and oncogenic virus while EBER1 and EBER2 are expressed in all cellular contexts of EBV latency. Nonetheless, these results must be taken with caution, as no change in AS were validated in this study.

Oncogenic viruses have a special relationship with host‐cell AS, as cancer cells present dysregulated AS profiles (David & Manley, 2010). In the context of cancer, it was demonstrated that EBV‐positive gastric carcinomas showed specific alteration in AS compared to EBV‐negative gastric carcinomas (Armero et al., 2017). The same conclusion was drawn with hepatitis B (HBV) and hepatitis C virus in hepatocellular carcinomas (Tremblay et al., 2016). In both cases, expression of a viral protein from those viruses possessing an oncogenic activity in cells (EBNA1 for EBV and HBx for HBV) induced changes in AS; nonetheless, high‐throughput RT‐PCR was used for validation on a limited number of AS events analyzed. Although these results seemed a little preliminary, a follow‐up study showed that the impact of the EBNA1 protein on cellular splicing seemed indirect, as the binding of EBNA1 to cellular RNAs does not directly leads to modifications in AS (Boudreault et al., 2019). Interestingly, the expression of E6 from HPV in HEK293T, which is also a potent oncoprotein, leads to a modulation of splicing in cellular genes (Xu et al., 2016). Still, the experimental design used was not optimal, since this study used a transient expression of E6 in HEK293T cells; a more relevant cell line and the usage of stable cell lines to ensure reproducible expression of E6 between cells would have been more relevant. As cancer cells present dysregulated AS profiles, an exciting possibility would be that viral oncoproteins drive the modulation of cellular AS towards a cancer‐like phenotype (David & Manley, 2010). To support this hypothesis, EBV‐induced carcinogenesis was shown by microarray analyses to alter the AS profiles of many AS events (Homa et al., 2013); however, more studies are needed to prove this hypothesis.

### 2.2 RNA viruses

Two recent studies have shown that mammalian reovirus, a member of the *Reoviridae* family of dsRNA viruses, can modulate the AS of the host‐cell following viral infection. The first one demonstrated that upon infection of L929 cells with the reovirus serotype 3‐Dearing strain (T3D) of reovirus, 240 AS events were modulated (Boudreault et al., 2016). The main strength of this study is that both AS‐PCR and mass spectrometry were used to validate the results, giving additional weight to the findings. In contrast, a later study by another group showed a limited effect of the T3D virus compared to the reovirus serotype 1‐Lang strain (T1L) (Rivera‐Serrano et al., 2017). This apparent discrepancy is likely attributable to variations in the actual amino acid sequences of the T3D strain used in different laboratories (Sandekian & Lemay, 2015). Interestingly, the second study further suggests that the interaction of the viral protein μ2 with the splicing factor SRSF2 in nuclear speckles is responsible for the modulation of cellular AS. The μ2 protein is an important structural protein that is partially located to the nucleus during infection and can bind RNA (Brentano, Noah, Brown, & Sherry, 1998; Kobayashi, Ooms, Chappell, & Dermody, 2009). Nevertheless, there was no ectopic expression of μ2 to demonstrate the ability of μ2 to modulate AS by itself, and there was no loss‐of‐phenotype experiment to prove that SRSF2 is involved in these changes. Overall, these data do not rule out the possibility that other viral determinants and/or mechanisms might be at play. Nonetheless, supporting the idea that μ2 is involved, the variant used in the first study possesses a proline at position 208 as in T1L, in contrast to the T3D variant of the second study that rather harbors a serine at this position (Sandekian & Lemay, 2015); this possibly explains the previously noted discrepancy in the effect on AS of T3D in the two studies. This proline to serine substitution in μ2 is already known to be involved in differences of IFN sensitivity/induction and morphology of viral factories between T3D and T1L (Irvin et al., 2012; Lanoie & Lemay, 2018; Parker, Broering, Kim, Higgins, & Nibert, 2002; Rivera‐Serrano et al., 2017; Zurney, Kobayashi, Holm, Dermody, & Sherry, 2009). In another study using a closely‐related avian reovirus, RNA‐sequencing of infected fibroblasts showed no change in cellular AS (Niu et al., 2017). It is still not clear if this avian strain is not able to directly modulate cellular AS or if the experimental design precluded the observation of such changes. Finally, in another more distant member of the *Reoviridae* family, the pathogenic rotavirus, the cytoplasmic splicing of the stress‐related factor XBP1 is altered following infection with some but not all of the rotaviral strains (Duarte et al., 2019). Interestingly, the NPS3 protein was identified as the primary determinant, but the splicing of XBP1 was always studied in the context of infection; ectopic expression of NSP3 should be tested to better understand all the determinants necessary to modulate splicing. Surprisingly, this is the only example of cytoplasmic splicing being modulated by viral infection, which is a relatively rare phenomenon and happens only in fewer than 30 genes (Buckley, Khaladkar, Kim, & Eberwine, 2014). Further studies will help to understand if modulation of nuclear splicing is conserved in the *Reoviridae* family, if other *Reoviridae* also modulates cytoplasmic splicing, and what are the molecular determinants behind those changes.

Studies have shown that RNA viruses from numerous other families are also able to trigger the modulation of host‐cell AS, which seems to indicate that viral modulation of cellular AS is a somewhat widespread phenomenon during virus–host interactions. Foot‐and‐mouth disease virus (FMDV), a *Picornaviridae,* often leads to persistence in infected cloven‐footed animals, but the molecular mechanisms allowing persistence of the virus remain unclear. Interestingly, cells that were adapted to enable persistent FMDV infection showed changes in cellular AS compared to non‐adapted cells (Han et al., 2018). For example, adapted cells presented changes in the AS of the splicing factor hnRNP A2B1, and this suggests that cellular AS could have a functional role in virus replication and persistence mechanisms. However, their validation was limited to 4 genes in RT‐PCR, and AS profiles in cultured cells can vary with passaging, which restrains the conclusion one can emit from this study. The recent outburst of interest in Zika virus, a *Flaviviridae* causing microcephaly in newborns and the Guillain‐Barré syndrome, spawned interest in changes induced by this virus following viral infection. Although it is solely transcriptomic, a study showed dysregulated AS following infection with this virus using RNA‐Seq (Hu et al., 2017). Similar results were shown following Dengue virus (DV) infection; in this case, an extensive PCR validation screen confirmed 32 AS events modulated by DV (Sessions et al., 2013). The demonstration about the ability of the NS5 protein of DV to modulate AS (see Section 3.4) and the transcriptomic studies altogether suggest that other *Flaviviridae* could have the same impact on cellular AS. It was recently concluded that reticuloendotheliosis virus, a retrovirus causing immunosuppression and cancer in avian species, trigger changes in AS of 859 cellular AS events (Gao et al., 2018). Nevertheless, the implications of these findings are limited since the authors focused their analyses on genes with AS that were differentially expressed following infection and never analyzed the modulation of AS per se, which is a major drawback of their study. Retroviruses, which integrate their genomes into the host cell DNA, present a particular case since they have a special relationship with host‐cell AS: the introduction of exogenous viral sequences containing splicing‐regulating signals could drive the production of aberrantly spliced transcripts. Integration of the viral genome of lentiviruses inside the genetic material of the host cell was demonstrated to induce aberrant splicing at the integration site, by bringing newly transcribed splice sites in pre‐mature RNAs (Cesana et al., 2012; Moiani et al., 2012). Another study, based on microarray chips, which are less efficient than RNA‐Sequencing for quantifying AS, showed that normal and malignant CD4^+^ T‐cells infected with HTLV‐1, an oncogenic retrovirus, display multiple alternate exon usage events as compared to non‐infected CD4^+^ T‐cells (Thénoz et al., 2014). Finally, in the case of influenza A virus (IAV), infection was also recently demonstrated to modulate the AS of 4 AS events which are co‐regulated by hnRNP K and NS1‐BP (Thompson et al., 2018). Moreover, laboratory and seasonal strains differ in the splicing modulation they induce (Fabozzi et al., 2018). Although the validation of this study was limited to three AS events (*TBK1*, *IFI35,* and *DDIT3*), it is reminiscent of the case of reovirus in which different strains have different impacts on AS, as discussed above.

### 2.3 Plant viruses

Although less studied, evidence for the involvement of plant viruses in the modulation of AS during virus–host interactions are emerging. For example, the Panicum mosaic virus and its satellite virus both trigger changes in AS in *Brachypodium distachyon* following infection (Mandadi & Scholthof, 2015). Interestingly, novel intron‐retaining variants of *SCL33*, a serine/arginine‐rich splicing factor, were identified and modulated by these two viruses. Moreover, infection of the hot pepper (*Capsicum annuum L*., an increasingly important crop worldwide) with cucumber mosaic virus leads to changes in the host AS (Zhu et al., 2018), and the bean common mosaic virus is also able to trigger alterations in host AS that seems to be strain‐specific (Martin et al., 2016). Except for *SCL33*, all these studies were solely transcriptomic and were limited by the annotations of AS in plants, which are still incomplete. Although the goal of the current review is to focus on viruses, it should be noted that dysregulation of AS was also observed in a tomato model of infection by the Potato Spindle Tuber Viroid (PSTVd), which is a self‐replicating RNA molecule (Zheng et al., 2017). As viroids do not encode for any proteins, it is quite intriguing how PSTVd infection alters AS. Although there was no RT‐PCR validation of changes in AS, recent studies showed that PSTVd can interact with RPL5 to modulate the splicing of TFIIIA, enhancing the levels of TFIIIA‐7ZF, an isoform essential for PSTVd replication, which suggests a potential mechanism for the modulation of AS (reviewed in Dissanayaka Mudiyanselage, Qu, Tian, Jiang, & Wang, 2018). In conclusion, novel high‐throughput approaches such as RNA‐Seq have allowed scientists to probe the transcriptome of infected cells extensively. These experiments have revealed the first hints at changes in cellular AS following infection with numerous virus from different families, a concept that scientists have only started to grasp.

## 3 MECHANISM‐DRIVEN APPROACHES

**Figure 3:**
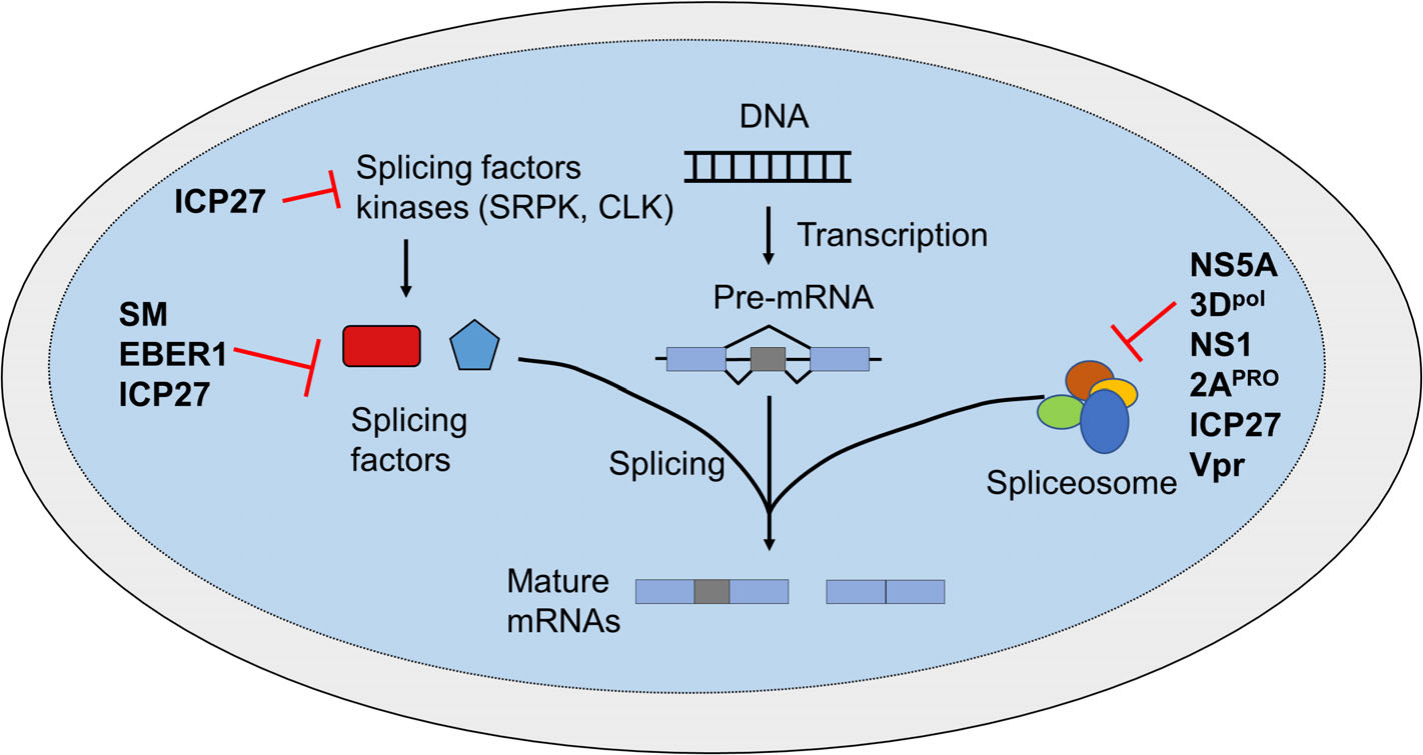
Schematic representation of the mechanisms of action for viral products that are potent modulators of cellular AS. NS5 from dengue virus, 3D^POL^ from picornavirus, NS1 from influenza virus, 2A^PRO^ from poliovirus, Vpr from HIV‐1 and ICP27 from *Herpesviridae* were all shown to interact with the spliceosome and inhibits the splicing reaction. SM and EBER1 from Epstein–Barr virus and ICP27 from herpes‐simplex virus 1 interact with splicing factors, and ICP27 is also able to interact with kinases that phosphorylate splicing factors. In the case of ICP27 which appears at numerous places in this figure, the mechanism of action sufficient to trigger a change in cellular AS is still not clear

Although interesting, these transcriptomic studies only depict the global portrait of the changes in cellular AS, with little insights into the mechanistic operating behind those changes. However, other studies have delved deeply into the characterization of viral products that are potent modulators of AS and their mechanism of action. The viral protein that has been the most studied is probably ICP27, an HSV protein which is well‐known as a potent AS inhibitor. Other examples that will be presented for DNA viruses include the EBV SM and EBER1 proteins. Then RNA viruses will be addressed, by discussing the RNA‐dependent RNA polymerase (RdRp) NS5 and 3Dpol from DV and picornavirus, the influenza virus NS1, poliovirus 2A^PRO^ and HIV‐1 Vpr. A summary of their mechanisms of action on cellular AS is depicted in Figure 3. and relevant information about this section is outlined in Table 2.

**Table 2:** Viral proteins involved in modulating the splicing machinery

Viral determinant	Virus	Family	Genome	Mechanism	Cellular genes with alternative splicing modification	References
**ICP27**	HSV‐I	Herpesviridae	dsDNA	Redistribution of snRNP Interaction with SRSF2, SRSF3 ICP27 interaction with SF3B2 Interaction with SRPK1 leading to hypophosphorylation of SR proteins	–	Hardy and Sandri‐Goldin (1994); Lindberg and Kreivi (2002); Phelan, Carmo‐Fonseca, McLaughlan, Lamond, and Clements (1993); Sandri‐Goldin, Hibbard, and Hardwicke (1995); Bryant, Wadd, Lamond, Silverstein, and Clements (2001); Sciabica (2003)
	HSV‐II			Direct interaction of ICP27 with PML pre‐mRNA	PML	Nojima et al. (2009)
	Herpesvirus saimiri			Redistribution of SRSF2	–	Cooper et al. (1999)
	Marek's disease virus			Interaction with SR proteins	chTERT	Amor et al. (2011)
**ORF57**	KSHV	Herpesviridae	dsDNA	Interaction with spliceosomal snRNP	–	Majerciak et al. (2008)
**SM**	Epstein–Barr virus	Herpesviridae	dsDNA	SM interaction with STAT1 pre‐mRNA; displacement of SRSF1 and recruitment of SRSF3	STAT1	Verma and Swaminathan (2008), Verma, Bais, Gaillard, and Swaminathan (2010)
**EBER1**	Epstein–Barr virus	Herpesviridae	dsDNA	Interaction with AUF1/hnRNP D	–	Lee, Pimienta, and Steitz (2012)
**NS5**	Dengue virus	Flaviviridae	(+)ssRNA	NS5 interaction with CD2BP2 and DDX23 from the U5 snRNP particle	ZNF35, CASP8, MXA, etc.	Maio et al. (2016)
**3D** ^**POL**^	EV71	Picornaviridae	(+)ssRNA	3D^POL^ interacts with Prp8 to block the second catalytic step of the splicing reaction	PIP85a, β‐globin, NCL	Liu et al. (2014)
**NS1A**	IAV	Orthomyxoviridae	(−)ssRNA	Binding to U6 snRNP, thus blocking the U6‐U4 interaction. SRSF2 relocalization in the nucleus Interaction with UAP56	TP53	Fortes, Beloso, and Ortín (1994); Fortes, Lamond, and Ortín (1995); Lu, Qian, and Krug (1994); Qiu, Nemeroff, and Krug (1995); Chiba, Hill‐Batorski, Neumann, and Kawaoka (2018); Dubois et al. (2019)
**2A** ^**pro**^	Poliovirus	Picornaviridae	(+)ssRNA	2A^pro^ blocks the second catalytic step of the splicing reaction	FAS, FGFR2 and MINX	Álvarez, Castelló, Carrasco, and Izquierdo (2011)
**Vpr**	HIV	Retroviridae	ssRNA‐RT	Vpr interaction with SF3B2 which block SF3B2‐SF3B4 interaction	–	Kuramitsu et al. (2005); Hashizume et al. (2007)

### 3.1 ICP27

The immediate‐early infected cell protein 27 (ICP27, also known as EI63) from the double‐stranded DNA virus HSV‐1 is known to be an important regulatory protein required for productive viral infection and expression of late viral genes (Sacks, Greene, Aschman, & Schaffer, 1985; Sandri‐Goldin & Mendoza, 1992). Moreover, this protein also inhibits splicing in the host cell during infection at early stages of spliceosome assembly (Hardy & Sandri‐Goldin, 1994; Lindberg & Kreivi, 2002). Interestingly, these results were obtained through in vitro splicing assays and thus might not adequately represent *in cellulo* conditions. ICP27 causes redistribution of small nuclear ribonucleoprotein particles (snRNP) to a more punctuate distribution (Phelan et al., 1993). Nonetheless, it was later shown that this redistribution and the interaction with SRSF2, requiring the C‐terminal repressor region of ICP27, correlates with the splicing inhibition but is not sufficient to induce this phenotype (Sandri‐Goldin et al., 1995). In this case, the splicing was studied *in cellulo*, further confirming the ability of ICP27 to inhibit splicing. The redistribution of the splicing factor SRSF2 seems to be conserved, as it also occurs with the ICP27 protein of *herpesvirus saimiri* (HVS; Cooper et al., 1999). ICP27 modulation of splicing was later demonstrated to require its interaction with SF3B2, an essential pre‐mRNA splicing factor, through its C‐terminal region (Bryant et al., 2001). In contrast, another study showed that ICP27 interacts with SR proteins such as SRSF3 and decreases their phosphorylation status (Sciabica, 2003). This under‐phosphorylation of SR proteins necessitates the ICP27 interaction with the SR protein kinase 1 (SRPK1), leading to its relocalization to the nucleus, and was shown to be sufficient to inhibit splicing. Caution must be taken regarding these results, as the experiments conducted to investigate the modulation of splicing were solely in vitro splicing experiments that might not completely recapitulate the complexity of the regulation *in cellulo*. To this date, the precise molecular determinants of ICP27 inhibition of splicing are still not precise, although the protein has been the subject of multiple reviews (Sandri‐Goldin, 1994; Sandri‐Goldin, 1998, 2008; Smith, Malik, & Clements, 2005). Many ICP27 homologs share biological features reminiscent of ICP27. In herpes simplex virus type 2 (HSV‐2), ICP27 changes the AS of the promyelocytic leukemia (*PML*) gene by inducing the retention of the intron 7a in the pre‐mRNA, leading to a switch from isoform PML‐II to PML‐V (Nojima et al., 2009). Interestingly, PML‐II favors viral replication as opposed to PML‐V which limits viral replication, suggesting that ICP27 might modulate cellular AS to restrict viral replication. In Marek's disease virus (MDV‐1), ICP27 interacts with SR proteins and inhibits the splicing of the *chTERT* gene (Amor et al., 2011). Finally, the ORF57 of the Kaposi's Sarcoma‐Associated Herpesvirus (KSHV) encodes a homolog of ICP27 which colocalizes with SRSF2 in nuclear speckles and interacts with the five spliceosomal snRNPs (U1, U2, U4, U5, and U6); this interaction might be indirect, as ORF57 seems to interact with nascent RNA, and RNAse treatments were not performed in the ORF57 pulldowns (Majerciak et al., 2008).

### 3.2 SM

The SM protein from EBV is a nuclear protein essential for EBV lytic replication through its RNA‐binding activity, allowing enhanced EBV gene expression by increasing EBV transcripts stability and nuclear export. It is a homolog of the previously discussed ICP27. Moreover, it also acts as a splicing factor on cellular transcripts through direct interaction, leading to the displacement of SRSF1 from the pre‐mRNA and the recruitment of SRSF3. This activity impacts the splicing of *STAT1*, for which SM favors a novel STAT1β isoform producing a protein acting as a dominant negative of the canonical STAT1α (Verma et al., 2010; Verma & Swaminathan, 2008). Because STAT1 has a vital role in the IFN signal transduction pathway, this modulation of *STAT1* splicing is presumed to impact the antiviral response, although it was not experimentally verified. The splicing of *STAT1* was the only example of SM‐mediated change in cellular AS.

### 3.3 EBER1

As previously discussed, the expression of EBER1 and EBER2 from EBV in cells lead to significant changes in the expression and the splicing of cellular genes (Pimienta et al., 2015, p. 1). Although the molecular mechanism for EBER2 is not known, evidence for EBER1 points towards its capacity to interact mainly with the p40 isoform of the AUF1/hnRNP D splicing factor (Lee et al., 2012). Nevertheless, no study directly looked at the ability of EBER1 alone to modulate cellular AS nor the involvement of AUF1/hnRNP D in this probable modulation of AS.

### 3.4 NS5

For the *Flaviviridae* family, the NS5 protein harbors the catalytic sites of RdRp, guanylyltransferase, and methyltransferase, allowing the production of fully‐capped mRNA during replication. Moreover, it is the only one of two proteins possessing enzymatic activity for this family of viruses, the other one being NS3 (Brand, Bisaillon, & Geiss, 2017). Recently, in an extensive study, it was demonstrated that NS5 from DV interacts with core components of the spliceosome, more precisely CD2BP2 and DDX23 from the U5 snRNP particle (Maio et al., 2016). This interaction led to a modulation of the splicing of cellular genes such as *ZNF35*, *CASP8*, and *MXA*. In the context of viral infection, increased intron retention was demonstrated during DV infection in cells and silencing specific U5 component also improved viral replication. The authors hypothesized that hijacking of the spliceosome by NS5 could allow a more favorable cellular environment for viral replication.

### 3.5 3D^POL^

As described for DV, the RdRp from picornavirus, more precisely Enterovirus 71 (EV71), is also able to localize to the nucleus. Once in the nucleus, it interacts with Prp8, one of the main components of the spliceosome, leading to the accumulation of the lariat form of the splicing intermediate due to a blockade of the second catalytic step (Liu et al., 2014). Genes that have their splicing modulated belongs to cell growth, proliferation, and differentiation pathways. It is interesting to underline that as previously discussed, persistently infected cells with another member of the *picornaviridae* family, MFDV, display AS modulation (Han et al., 2018). It will be interesting to know if the RdRp from MFDV is involved in those changes. Another mechanism involving the cleavage of Sam68 will be discussed in Section 4.1.

### 3.6 NS1

The NS1 protein from IAV is a crucial multifunctional protein for viral replication that suppresses the innate antiviral response. Moreover, NS1 is also a potent inhibitor of splicing. NS1 interacts with the spliceosome through its RNA‐binding domain, with the consequence of inhibiting the catalytic steps required for adequate splicing (Fortes et al., 1994; Lu et al., 1994). It was later shown that NS1 binds to specific regions of U6 snRNA. This binding blocks the interaction of U6 to both U2 and U4 snRNP and thus explains the inhibition of splicing (Qiu et al., 1995). These findings are somewhat surprising, as IAV necessitate the host splicing machinery to splice some of its RNA, notably the M1 segment coding for NS1. However, the M1 gene segment is still spliced in presence of NS1 despite the blockade on splicing, which suggests that the inhibition in splicing is specific for cellular RNA; further studies are required to confirm this and explain the observed phenomenon (Fortes et al., 1994; Lu et al., 1994). NS1 expression also changes the nuclear localization pattern of splicing factor SRSF2, both in the context of ectopic expression and of viral infection (Fortes et al., 1995). Recently, mass spectrometry analysis of protein–protein interaction of NS1 from both strains H1N1 and H7N9 showed enrichment in interacting partners involved in mRNA splicing and RNA processing (Kuo et al., 2018, 2016). Interestingly, the two proteins did not interact with the same cellular partners, which suggests that there might exist species‐specific modulation of AS, or that the identified proteins are the result of artificial noise from the experimental design. Moreover, it was also shown that NS1 interacts with UAP56, an helicase involved in pre‐mRNA splicing; the impact of this interaction is still not known (Chiba et al., 2018). As previously discussed, IAV‐infected cells present modulation in their AS, which make the NS1 protein an interesting model to study viral modulation of AS (Thompson et al., 2018). Finally, it was recently demonstrated that NS1 modulates the splicing of TP53 towards β and γ isoforms, which are pro‐viral factors in IAV infection (Dubois et al., 2019).

### 3.7 2A^PRO^

Poliovirus genome produces a single transcript that is translated into a long polyprotein that is processed by viral protease 2A^PRO^ and 3C^PRO^. Moreover, those proteins also cleave cellular proteins, leading to their degradation in infected cells. Surprisingly, the 2A^PRO^ protease is also an inhibitor of splicing, which was demonstrated for the *FAS*, *FGFR2* and *MINX* genes (Álvarez et al., 2011). Since in vitro splicing assay of the *MINX* gene showed accumulation of the first exon and the lariat product containing the unspliced second exon, it was concluded that 2A^PRO^ blocks the second catalytic step of the splicing reaction. Again, caution must be taken regarding in vitro experiments, as their results cannot be directly transferred *in cellulo*.

### 3.8 Vpr

Vpr, a small 96 amino acid protein from HIV‐1, was shown to inhibit the splicing reaction both in vitro and *in cellulo* (Kuramitsu et al., 2005). Later studies proved that this inhibition is dependent on the interaction with SF3B2, which blocks the essential interaction of SF3B2 with SF3B4 (Hashizume et al., 2007). Nonetheless, these results are difficult to reconcile with the biology of HIV replication, as this virus needs the cellular machinery to splice its RNA. Still, the authors have shown that some introns were not affected by this inhibition. If Vpr can mediate a blockade of splicing only on cellular RNA, as it might be the case for NS1, then further studies should eventually explain how this cellular‐specific blockade is possible.

## 4 MODULATION OF SPLICING FACTORS LOCALIZATION, LEVELS AND POST‐TRANSLATIONAL MODIFICATIONS BY VIRUSES

Other mechanisms can be at play during viral infections that do not necessitate direct interaction with the cellular splicing machinery but can also have an impact on cellular splicing. Cellular localization, post‐translational modifications, and levels of splicing factors can all affect the outcome of the splicing reaction (David & Manley, 2010; Shin & Manley, 2004; Twyffels, Gueydan, & Kruys, 2011). This strongly suggests that viruses could modulate cellular AS through these indirect mechanisms, and the following section will look further into the known examples for each of these mechanisms. An overview of viruses and splicing factors discussed is available in Table 3.

**Table 3:** List of viruses having a potential indirect effect on alternative splicing

Virus	Family	Genome	Splicing factors impacted	References
**Modulation of splicing factor localization**				
**EBV**	*Herpesviridae*	dsDNA	SRSF2, SRSF3, SON	Park and Miller (2018)
**Vesicular stomatitis virus**	*Rhabdoviridae*	(−)ssRNA	hnRNP A1, K and C1/C2 hnRNP M	Kneller, Connor, and Lyles (2009) Redondo, Madan, Alvarez, and Carrasco (2015)
**Poliovirus**	*Picornaviridae*	(+)ssRNA	HuR, TIA1/TIAR SRSF3 hnRNP M	Álvarez, Castelló, Carrasco, and Izquierdo (2013) Fitzgerald, Chase, Cathcart, Tran, and Semler (2013) Jagdeo et al. (2015)
**Coxsackievirus B3**	*Picornaviridae*	(+)ssRNA	SRSF3 hnRNP M	Fitzgerald et al. (2013) Jagdeo et al. (2015)
**Rhinovirus**	*Picornaviridae*	(+)ssRNA	SFPQ	Flather, Nguyen, Semler, and Gershon (2018)
**Sindbis virus**	*Togaviridae*	(+)ssRNA	HuR, hnRNP K	Barnhart, Moon, Emch, Wilusz, and Wilusz (2013)
**HIV**	*Retroviridae*	ssRNA‐RT	hnRNP A1	Monette, Ajamian, López‐Lastra, and Mouland (2009)
**Junin/DV**	*Flaviviridae*	(+)ssRNA	hnRNP A2, K	Brunetti, Scolaro, and Castilla (2015)
**MFDV**	*Picornaviridae*	(+)ssRNA	Sam68	Lawrence, Schafer, and Rieder (2012)
**HCV**	*Flaviviridae*	(+)ssRNA	HuR	Shwetha et al. (2015)
**Rotavirus**	*Reoviridae*	dsRNA	HuR, hnRNP C1	Dhillon et al. (2018)
**Modulation of splicing factor levels**				
**HCMV**	*Herpesviridae*	dsDNA	CstF‐64, PTB	Adair, Liebisch, Su, and Colberg‐Poley (2004)
**HPV**	*Papillomaviridae*	dsDNA	SRSF1, SRSF2. SRSF3. SRSF4	Klymenko, Hernandez‐Lopez, MacDonald, Bodily, and Graham (2016), McPhillips et al. (2004); Mole et al. (2009); Mole, Milligan, and Graham (2009)
**HIV**	*Retroviridae*	ssRNA‐RT	SRSF2, hnRNP A/B, hnRNP H SR proteins	Dowling et al. (2008) Fukuhara et al. (2006)
**Modulation of splicing factor post‐translational modifications**				
**HSV‐1**	*Herpesviridae*	dsDNA	SRSF3, SRSF5 (hyperphosphorylated)	Sciabica (2003)
**Adenovirus**	*Adenoviridae*	dsDNA	SR proteins (hypophosphorylated)	Kanopka et al. (1998)
**Vaccinia virus**	*Poxviridae*	dsDNA	SR proteins (hypophosphorylated)	Huang, Nilsson, Punga, and Akusjärvi (2002)
**HIV‐1**	*Retroviridae*	ssRNA‐RT	SRSF2 (hyperphosphorylated)	Kadri et al. (2015)
**Sindbis virus**	*Togaviridae*	(+)ssRNA	HuR (hypophosphorylated)	Dickson et al. (2012)

### 4.1 Modulation of splicing factors localization

Several viruses modulate the localization of splicing factors during infection. For example, vesicular stomatitis virus (VSV) infection leads to cytoplasmic accumulation of hnRNP A1, hnRNP K, and hnRNP C1/C2 through a mechanism that is dependent on the mRNA export factor RAE1 (Kneller et al., 2009). A study suggested that the Vesicular stomatitis virus M proteins are involved in this relocalization, at least for the splicing factor hnRNP H (Redondo et al., 2015). Moreover, the poliovirus 2A^pro^, besides being an inhibitor of splicing as seen in the previous section, also induces a selective nucleo‐cytoplasmic translocation of the splicing factors and RNA‐binding proteins HuR and TIA1/TIAR (Álvarez et al., 2013). This redistribution leads to the modulation of the splicing of the exon 6 of the *FAS* gene. SRSF3 and hnRNP M localization were also shown to be impacted by infection from *Picornaviridae* (Fitzgerald et al., 2013; Jagdeo et al., 2015). Recently, an MS‐based approach was used to look broadly at proteins re‐equilibrating from the nucleus to the cytoplasm during infection with a rhinovirus, which is another member of the *Picornaviridae* family (Flather et al., 2018). This study identified SFPQ, a splicing factor, as a pro‐viral factor that is relocalized to the cytoplasm following cleavage by the viral proteinase 3CD/3C. Furthermore, Sindbis virus also induces the relocalization of HuR and hnRNP K from the nucleus to the cytoplasm of infected cells (Barnhart et al., 2013; Burnham, Gong, & Hardy, 2007; Dickson et al., 2012). Interestingly, the mechanism for HuR depends on the viral RNAs, which contains HuR binding‐site in their 3’ UTR, and the phosphorylation of HuR (discussed in Section 4.3). This relocalization also induces changes in the splicing of known targets of HuR, such as *PCBP2* and *DST*. HuR seems to be a target of choice for viruses to affect relocalization, as HCV and rotavirus (later discussed) also induce its accumulation in the cytoplasm (Dhillon et al., 2018; Shwetha et al., 2015). More examples of splicing factors relocalization during viral infection have been described, such as hnRNP A1 during HIV infection (Monette et al., 2009), hnRNP A2 and hnRNP K during DV and Junìn virus infection (Brunetti et al., 2015), and Sam68 during MFDV infection (Lawrence et al., 2012). Finally, many viruses rely heavily on replication structure built by viral proteins for their replication cycle. In the case of rotavirus, a segmented dsRNA virus solely replicating in the cytoplasm, these structures are named viroplasms and have been shown to sequester hnRNPs and AU‐rich element‐binding proteins (ARE‐BPs) independently of RNA (Dhillon et al., 2018). The proteins sequestered, such as HuR and hnRNP C1, act as positive or negative regulator in viral progeny production. During lytic EBV infection, novel nodular structures composed of viral and cellular RNA splicing and export factors, termed VINORCs (for virus‐induced nodular structures), were recently discovered and were shown to colocalize with splicing factors such as SRSF2, SRSF3, SON, and NXF1 (Park & Miller, 2018). These examples underline the capacity of viruses to modulate AS by changing the cellular localization of splicing factors, and in cases where cellular splicing was not analyzed, suggest an impact at this level in the host cell. It also stresses the crucial importance not only to look at the cellular localization of splicing factors but also to monitor the impact of this localization (or relocalization) on cellular AS.

### 4.2 Modulation of splicing factors levels

Viral infections also trigger massive changes in the gene expression program of infected cells, and several viruses have been shown to modulate the levels of expression of splicing factors. For example, HCMV infection increases the expression of the cleavage stimulation factor 64 (CstF‐64) and PTB (Adair et al., 2004). Moreover, in the case of HIV, macrophages are an important viral reservoir, and it was found that HIV infection leads to an upregulation of the SR protein SRSF2 and a down‐regulation of hnRNP A/B and hnRNP H in the macrophages during the first weeks of infection. Around the peak of virus production, the trend is inverted: the level of SRSF2 decreases and the level hnRNP A/B and hnRNP H increases (Dowling et al., 2008). HIV infection of HEK293 cells also decreases the overall level/activity of SR proteins (Fukuhara et al., 2006). HPV replication is heavily dependent on the expression of some specific splicing factors, such as SRSF2 and SRSF3, at different times during infection. It appears that the virus has evolved to modulate the expression of these proteins (Klymenko et al., 2016; McPhillips et al., 2004; Mole, McFarlane, et al., 2009; Mole, Milligan, & Graham, 2009); this subject was reviewed extensively (Klymenko & Graham, 2012; McFarlane & Graham, 2010; Mole, Veerapraditsin, McPhillips, & Graham, 2006). Lastly, it was demonstrated that reovirus infection leads to transcriptional changes in the expression levels of splicing factors, with ESRP1 which has an epithelium‐specific role being the most overexpressed following infection (Boudreault et al., 2016). Although the impact on cellular AS may be limited, these studies underline that viruses can alter the expression levels of splicing factors throughout infection.

### 4.3 Modulation of splicing factors post‐translational modifications

Post‐translational modifications, such as phosphorylation, have been known to influence the activity of splicing factors. Various studies have demonstrated that viral infection can also induce changes in the post‐translational modification profiles of splicing factors. For example, SR proteins from adenovirus‐infected cells are hypophosphorylated, which renders them inactive as enhancers or splicing repressors. This dephosphorylation requires the interaction of the adenovirus protein E4‐ORF4 with the protein phosphatase 2A (PP2A) and impacts SRSF1, SRSF2, SRSF4, SRSF5, and SRSF6 (Kanopka et al., 1998). Moreover, E4‐ORF4 also interacts directly with SRSF2 and SRSF9, mainly with the hyperphosphorylated forms (Nilsson et al., 2001). The same group observed a similar hypophosphorylation and inactivation of SR proteins in vaccinia virus‐infected cells, with possible involvement of the viral dual‐specificity protein phosphatase VH1 (Huang et al., 2002). Interestingly, the extent of dephosphorylation was greater in vaccinia infection that in adenovirus infection, which seems to correlate with the absence of introns in vaccinia genes compared to adenoviral genes. In this case, as seen before with the studies on the expression of splicing factors during infection, the biological impact on cellular AS was not assessed. The HIV‐1 Tat protein was shown to modulate *Tau* AS, by upregulating DYRK1A kinase which leads to increased phosphorylated SRSF2 (Kadri et al., 2015). As previously discussed, infection with Sindbis virus causes relocalization of HuR to the cytoplasm; this relocalization is dependent on the dephosphorylation of the protein (Dickson et al., 2012). Moreover, ICP27 also interacts with SRPK1, leading to hypophosphorylation of splicing factors (Sciabica, 2003). A similar activity was demonstrated for the HPV protein E1^E4 (Prescott et al., 2014). Although only Tat was shown to influence cellular AS, acting on the phosphorylation level of splicing factors is another exciting mechanism by which viruses could potentially modulate the cellular splicing machinery.

## 5 BIOLOGICAL RELEVANCE OF CELLULAR AS MODULATION

Upon viral infection, a tug of war between the virus and the cell is triggered with the ultimate consequence of allowing or restricting viral replication. On the one hand, viruses try to hijack essential cellular components such as DNA polymerases and ribosomes, to replicate and block the innate immune response triggered by the IFN pathway. On the other hand, the cells try to mount an effective antiviral response and limit virus replication, for example by shutting down translation. During viral infection, modulation of cellular AS could be directly caused by viral proteins or RNA, and/or could be triggered by the cell as a self‐defense mechanism to counteract the virus. Moreover, this response could also be driven through the IFN pathway and could be triggered in non‐infected cells by soluble antiviral factors to prime these cells before viral infection. To this date, the functional importance of viral modulation of AS during viral infection is still not evident. However, the examples of NS5A from DV, 3D^pol^ from EV71, and 2A^PRO^ from poliovirus seem to point towards viruses being able to modulate cellular AS directly, probably as ways to favor their replication (Table 4) (Álvarez et al., 2011, 2013; Liu et al., 2014; Maio et al., 2016). Interestingly, splicing factors often act as host factors enhancing or restricting viral replication, underlining their important role in virus biology. For example, hnRNP C1/C2 during DV infection (Dechtawewat et al., 2015), hnRNP K during DV/Junìn virus infections (Brunetti et al., 2015), hnRNP D during West Nile virus infection (Friedrich et al., 2014), hnRNP M during picornaviruses infections (Jagdeo et al., 2015), SART1 and HuR during HCV infection (Lin et al., 2015, p. 1; Shwetha et al., 2015; Zhao et al., 2012), PRP19 during IAV H1N1 infection (Kuo et al., 2016, p. 19) and Sam68 during FMDV infection (Lawrence et al., 2012, p. 68) all acts as positive regulators of viral replication. The opposite effect can also be seen, as in the context of reovirus replication, gene silencing of SRSF2, an important splicing factor that interacts with the reovirus protein μ2, enhances the replication of this virus (Rivera‐Serrano et al., 2017). However, the involvement of splicing factors in virus replication does not necessarily indicate that AS modulation of the host cell transcriptome directly stimulates or inhibits viral replication, and further studies will help our understanding of this potential dual role of splicing factors. Nevertheless, infection of cells with HSV‐1 favors a spliced variant of MxA, a potent antiviral factor, that enhances HSV‐1 replication instead of limiting it, and this is perhaps the best evidence of a virus benefiting from modulating cellular AS (Ku et al., 2011). The potential of abrogating the IFN response and IFN‐stimulated effector through AS is important and could serve as another mechanism for viruses to evade the antiviral state triggered by infection (Chauhan, Kalam, Dutt, & Kumar, 2019). Still, a study that looked at both the expression and AS following infection revealed that alterations in AS are not happening preferentially in overexpressed genes, which are usually ISG linked to the cellular antiviral state (Boudreault et al., 2016). In a study about new therapies directed against HIV‐1 and respiratory syncytial virus, specific inhibition using a morphilino oligonucleotide against the long isoform of the molecular chaperone MRJ leads to a decreased replication of these two viruses (Ko et al., 2019). Another biological relevant example for virus–host interaction is ICP27 of HSV‐2, as previously discussed; the expression of this protein leads to a switch from a PML isoform favoring viral replication to one which is limiting the replication of HSV‐2 (Nojima et al., 2009). Although minimal, examples about MxA, MRJ, and PML point towards a potential direct benefit for viruses to modulate the AS landscape of the host cell to better replicate, or to limit their replication under a certain threshold. It is also noteworthy to underline that transcriptomic studies reveal the global portrait of the RNA splicing landscape of the host cell independently of the cause of these changes, and the possibility that the cell could trigger AS change to defend itself should still be considered. In fact, it is highly possible that transcriptomic studies describe modulations that are caused both by viruses and the host cell. Lastly, it is important to remember that if the modulation of cellular AS presents an advantage for viruses, infected cells might have evolved a countermeasure to restrict this mechanism, which emphasizes the complexity of adequately mapping the specific cellular and viral determinants of these changes in the context of virus–host interaction.

**Table 4:** List of viruses that modulate cellular AS with a known mechanism, and the cellular genes impacted

Virus	Family	Genome	Cellular genes with alternative splicing modification	Mechanism of modulation/determinant	References
Herpes‐simplex virus II	*Herpesviridae*	dsDNA	PML	ICP27	Nojima et al. (2009)
Marek's disease virus	*Herpesviridae*	dsDNA	chTERT	Probable interaction of ICP27 with SRSF3	Amor et al. (2011)
Epstein–Barr virus	*Herpesviridae*	dsDNA	STAT1	SM protein displaces SRSF1 from pre‐mRNA and recruits SRSF3	Verma et al. (2008) Verma et al. (2010)
Human cytomegalovirus	*Herpesviridae*	dsDNA	CAST, MYO18A	Overexpression of CPEB1 following infection	Batra et al. (2016)
Sindbis virus	*Togaviridae*	(+)ssRNA	PCBP2 DST	Viral RNAs containing HuR binding‐site in their 3’ UTR induces the relocalization of HuR to the cytoplasm	Barnhart t al. (2013)
tDengue virus	*Flaviviridae*	(+)ssRNA	ZNF35, CASP8, MXA, etc.	NS5 interacts with CD2BP2 and DDX23 from the U5 snRNP particle	Maio et al. (2016)
EV71	*Picornaviridae*	(+)ssRNA	PIP85a, β‐globin, NCL	3D^POL^ interacts with Prp8 to block the second catalytic step of the splicing reaction	Liu et al. (2014)
Poliovirus	*Picornaviridae*	(+)ssRNA	FAS, FGFR2 and MINX	2A^pro^ blocks the second catalytic step of the splicing reaction.	Álvarez et al. (2011)
			FAS	2A^pro^ induces a selective nucleo‐cytoplasmic translocation of HuR and TIA1/TIAR	Álvarez et al. (2013)
Influenza virus A	Orthomyxoviridae	(−)ssRNA	TP53	Interaction of NS1 with CPSF4	Dubois et al. (2019)

The state of knowledge is still too sparse to identify common cellular mRNA targets which have their AS modulated by numerous viruses. Nonetheless, a common theme is starting to emerge in which cellular genes involved in mRNA processing, including genes linked to mRNA maturation, degradation, and notably, splicing appear to have changes in AS following viral infection (Boudreault et al., 2016; Fabozzi et al., 2018; Han et al., 2018; Hu et al., 2017, 2016; Rivera‐Serrano et al., 2017). Whether this indeed constitutes a targeted modulation of AS still remains to be established. Nonetheless, an attractive hypothesis would be that viruses could preferentially target the AS of genes involved in splicing, which would ultimately lead to abnormal mRNA isoform expression for those genes. This would further dysregulate cellular splicing by altering the normal balance of protein isoforms, changing their finely regulated activity and disturbing the splicing machinery.

## 6 CONCLUSIONS AND PERSPECTIVES

The advent of affordable high throughput sequencing techniques has allowed scientists to delve deeply into the transcriptomic changes happening during virus–host interactions. These studies, combined with mechanistic ones, have allowed the discovery and characterization of AS changes following viral infections in different contexts. Although this field of study is still relatively recent, the accumulating evidence point towards widespread changes in cellular AS following infections by viruses. A better understanding of how and why viruses modulate cellular AS will surely help to gain a better global understanding of virus–host interaction and the role of cellular AS in this interplay. Recently, it was shown that the splicing factor SRSF2 limits HSV‐1 oncolysis in breast cancer cells, and it also negatively regulates the replication of other promising oncolytic viruses such as reovirus (Rivera‐Serrano et al., 2017; Workenhe, Ketela, Moffat, Cuddington, & Mossman, 2015). Understanding of the role of cellular AS in viral replication could thus help the design of better oncolytic viruses exploiting the dysregulated AS landscape typical of cancer cells (David & Manley, 2010). Moreover, as previously discussed, numerous splicing factors are involved in viral replication, either as positive or negative factors. Potential new antiviral drugs targeting host cell splicing factors have emerged, and a better understanding of the role of cellular AS could help the design of more potent or safer antivirals (Fukuhara et al., 2006; Shkreta et al., 2017). All these fields will benefit from a better understanding of the role of cellular AS modification during viral infection, a phenomenon we have only just begun to investigate.

## CONFLICT OF INTEREST

The authors have declared no conflicts of interest for this article.

## RELATED WIREs ARTICLE

Unconventional RNA‐binding proteins step into the virus–host battlefront
